# Multichamber Involvement of Metastatic Cardiac Melanoma

**DOI:** 10.3390/diagnostics12030587

**Published:** 2022-02-25

**Authors:** Maria S. Bonou, Panagiotis Diamantopoulos, Sofia Mavrogeni, Chris J. Kapelios, John Barbetseas, Helen Gogas

**Affiliations:** 1 Department of Cardiology, Laiko General Hospital, 17, Agiou Thoma Street, Goudi, 11527 Athens, Greece; chriskapel@hotmail.com (C.J.K.); jbnv@otenet.gr (J.B.); 2 First Department of Internal Medicine, Laikon General Hospital, National and Kapodistrian University of Athens, 15772 Athens, Greece; pandiamantopoulos@gmail.com (P.D.); helgogas@gmail.com (H.G.); 3 Department of Cardiology, Onassis Cardiac Surgery Center, 17674 Athens, Greece; sophie.mavrogeni@gmail.com

**Keywords:** melanoma, cardiac metastases, multimodality imaging

## Abstract

A 30-year-old man with a history of an in-situ melanoma of the forehead was referred for cardiac evaluation because of tachycardia and elevated levels of serum troponin. The transthoracic echocardiogram revealed multiple masses attached to the walls of both ventricles and the right atrium (RA). A large mass was occupying almost one third of the right ventricle (RV), resulting in reduction of the end-diastolic RV volume and tachycardia. A cardiac magnetic resonance imaging confirmed multifocal myocardial infiltration and intracavitary masses and excluded the presence of thrombus in any of the cardiac chambers. Diffuse metastatic involvement in the liver, the spleen, and the brain by computed tomography precluded surgical management. Being BRAF-unmutated, the patient was initially treated with a combination of nivolumab and ipilimumab. One month later, the cardiac metastases in RA and left ventricle were unchanged on echocardiogram, while the tumor in RV was enlarged occupying the majority of the chamber, resulting in further reduction of the cardiac output and tachycardia. The treatment was changed to a combination of dacarbazine and carboplatin, but the patient eventually died two months later. Heart is not a common metastatic site of melanoma and cardiac involvement is usually clinically silent making ante mortem diagnosis difficult. Multimodalidy imaging plays a pivotal role in the diagnostic work up. Cardiac melanoma metastases indicate an advance stage disease with poor prognosis.

**Figure 1:**
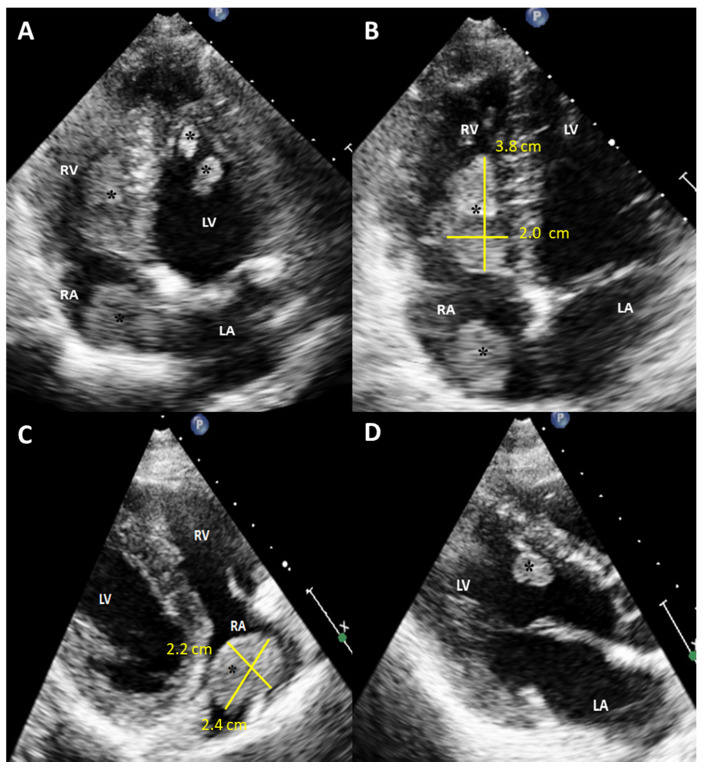
Initial scan: Transthoracic echocardiogram images, four chamber apical view (**A**,**B**) and parasternal long axis view (**C**,**D**). Multiple masses in the cardiac chambers are marked with black asterisks. A 30-year-old Caucasian man with a history of an in-situ melanoma of the forehead, which was surgically excised four years earlier, was referred for cardiac evaluation because of tachycardia and elevated levels of serum troponin. The patient had presented three months ago to the oncology clinic, with dry cough and pigmented skin nodules on the left shoulder, left forearm, chest, and forehead. Abdominal and brain computed tomography (CT) scans showed several metastatic lesions in the liver, the spleen, and the brain, while the histologic evaluation of one of the skin lesions revealed metastatic melanoma. Being BRAF-unmutated, the patient was initially treated with a combination of nivolumab and ipilimumab but due to grade 3 colitis, the treatment was replaced by a combination of dacarbazine and carboplatin. During clinical examination he was hemodynamically stable and his electrocardiogram showed sinus tachycardia at 120 beats per minute. The echocardiogram revealed multiple masses attached to the walls of both ventricles and the right atrium (RA) while no mass was detected in the left atrium (LA) (**A**). A large intracavitary mass (3.8 cm × 2.0 cm) was occupying almost one third of the right ventricle (RV) while a lobular right atrial nodule (2.4 cm × 2.2 cm) was attached to the posterior wall of the RA by a small peduncle (**B**,**C**). Additionally, a small mass in the left ventricle (LV) was attached to the interventricular septum (**D**). The end-diastolic RV volume was severely reduced by the mass, resulting in compensatory tachycardia, while there was no significant tricuspid valve inflow obstruction. The biventricular function was mildly reduced. A cardiac magnetic resonance (CMR) imaging confirmed the multifocal myocardial infiltration and the intracavitary masses and excluded the presence of thrombus in any of the cardiac chambers (Figure 2). Diffuse metastatic involvement precluded surgical management. One month later the cardiac metastases in RA and LV were unchanged on echocardiogram, while the tumor in RV was enlarged occupying the majority of the chamber, resulting in further reduction of the cardiac output and tachycardia (Figure 3, Supplemental Video S1). The treatment was changed to a combination of dacarbazine and carboplatin, but the patient eventually died two months later. Our case illustrates a rarity of metastasis of a malignant melanoma of to the heart providing useful information for doctors in training and practitioners. Melanoma is a skin malignancy with increasing incidence during the last decades and one of the most common cancers in young adults [1]. While it is responsible for less than 5% of all cutaneous malignancies, it is potentially lethal accounting for the majority of skin cancer deaths. Although, cardiac melanoma metastases are rare, with multichamber involvement being even rarer, there is a higher propensity of cardiac metastases typically by a hematogenous route compared with other tumors. However, despite that cardiac involvement is present up to 50% based on autopsy findings, including myocardial or pericardial infiltration, pericardial fluid, and intracavitary masses, metastases to the heart are usually clinically asymptomatic or present with nonspecific symptoms including cough, tachycardia, and dyspnea, making ante mortem diagnosis and treatment much more difficult [2,3]. Multimodalidy imaging, such as echocardiography, cardiac CT, but mostly, CMR due to its ability to differentiate tumor from thrombus, plays a pivotal role in the diagnostic work up [3,4]. Transthoracic and transesophageal echocardiography provide useful information about the morphology, the location and the vascularity of the tumor using contrast agent, as well as cardiac anatomy and physiology. Although, several morphologic features have been described on CT or CMR to differentiate benign from malignant cardiac lesions, their specificity are rather low. Contrast-enhanced CT is helpful in tumor staging because of its ability to detect extracardiac metastases, while a more accurate evaluation of the size of the tumor and its relationship to the adjacent tissues can be extracted by 3D reconstructed images. Furthermore, CT in conjunction with positron emission tomography (PET) enables both locating the tumor and providing information about the metabolic activity of the cardiac masses. PET can differentiate benign from malignant cardiac tumors, with the latter showing high 18F-fluorodeoxyglucose uptake. Lastly, tissue characterization and myocardial infiltration can be provided on T1 and T2 weighted and late gadolinium enhancement CMR images. Cardiac melanoma is usually demonstrated hyperintense on T1 and hypointense on T2-weighted imaging compared to the adjacent myocardium. Melanin-rich melanomas and mitral annular calcification show relatively short T1 times [5]. Conversely, thrombi and myxomas show intermediate and relatively long T1 times, respectively, while pericardial cyst shows the longest T1 time. The relatively low T1 values (~700 ms) of the melanin-rich metastatic foci on the corresponding T1 map can identify the myocardium studded with tumor. Metastases to the heart from melanoma indicate an advanced stage disease with poor prognosis and are typically accompanied by widespread metastases in other organs such as the liver, the lung, and the brain. In these cases, chemotherapy and immune checkpoint inhibitors should be considered as first-line therapy, while in BRAF-mutated cases, targeted therapy is also an option. However, in highly selected cases with solitary cardiac metastasis, if complete surgical resection of the cardiac mass with tumor-free margins from the atria or the ventricles is feasible, surgery can be considered as a therapeutic option, with survival up to one year in some case reports [6]. Nevertheless, the benefit outcome from these surgical procedures remains unknown due to the lack of longitudinal data.

**Figure 2:**
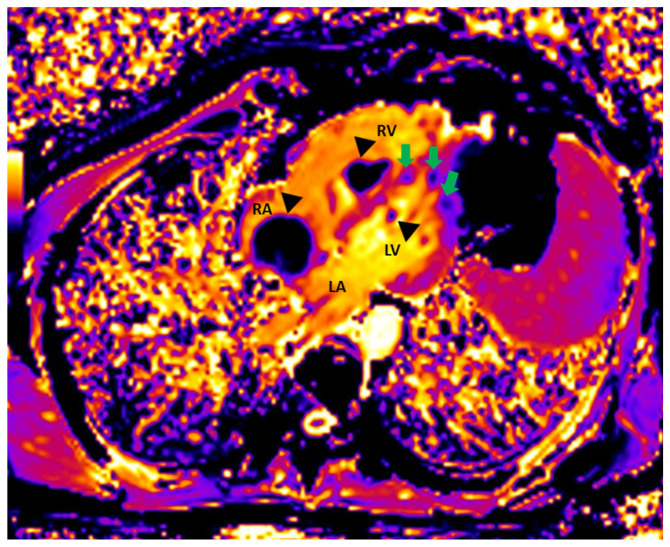
Cardiac magnetic resonance: Native T1 Image showing metastases in RA, RV, and LV (black arrrowheads). The lateral wall of LV and the apical interventricular septum are also infiltrated by metastatic lesions (green arrows).

**Figure 3:**
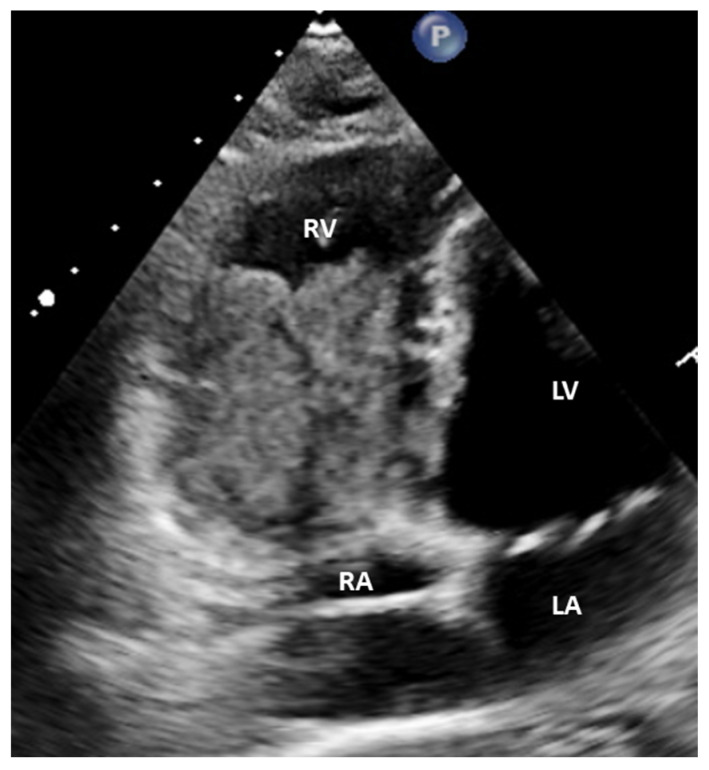
Echocardiogram, modified four chamber view: The tumor in RV has been enlarged occupying the majority of the chamber.

## Supplementary Materials

The following supporting information can be downloaded at: https://www.mdpi.com/article/10.3390/diagnostics12030587/s1, Video S1. Apical modified 4-chamber view on transthoracic echocardiography: The tumor in RV has been enlarged occupying the majority of the chamber while the tumor in RA is unchanged.
